# Highly sensitive dual mode electrochemical platform for microRNA detection

**DOI:** 10.1038/srep36719

**Published:** 2016-11-08

**Authors:** Pawan Jolly, Marina R. Batistuti, Anna Miodek, Pavel Zhurauski, Marcelo Mulato, Mark A. Lindsay, Pedro Estrela

**Affiliations:** 1 Department of Electronic & Electrical Engineering, University of Bath, Bath BA2 7AY, United Kingdom; 2 Department of Physics, University of São Paulo, 14040-901, Ribeirão Preto, SP, Brazil; 3 Department of Pharmacy & Pharmacology, University of Bath, Bath BA2 7AY, United Kingdom

## Abstract

MicroRNAs (miRNAs) play crucial regulatory roles in various human diseases including cancer, making them promising biomarkers. However, given the low levels of miRNAs present in blood, their use as cancer biomarkers requires the development of simple and effective analytical methods. Herein, we report the development of a highly sensitive dual mode electrochemical platform for the detection of microRNAs. The platform was developed using peptide nucleic acids as probes on gold electrode surfaces to capture target miRNAs. A simple amplification strategy using gold nanoparticles has been employed exploiting the inherent charges of the nucleic acids. Electrochemical impedance spectroscopy was used to monitor the changes in capacitance upon any binding event, without the need for any redox markers. By using thiolated ferrocene, a complementary detection mode on the same sensor was developed where the increasing peaks of ferrocene were recorded using square wave voltammetry with increasing miRNA concentration. This dual-mode approach allows detection of miRNA with a limit of detection of 0.37 fM and a wide dynamic range from 1 fM to 100 nM along with clear distinction from mismatched target miRNA sequences. The electrochemical platform developed can be easily expanded to other miRNA/DNA detection along with the development of microarray platforms.

MicroRNAs (miRNAs) are a class of noncoding genes that are transcribed as RNA sequences of around 22 nucleotides long^1^. More than twenty years after the first article about miRNA, there have been many reports on the role of miRNAs in gene expression and regulation^2^,^3^. Many reports have shown their importance in various biological processes in the human body including regulation of target gene^4^, cellular proliferation and differentiation^5^, cell death^6^, translational and transcriptional regulation of their expression^7^. It was only in the last decade that miRNAs have been identified for their importance in human diseases such as cancer^8^,^9^,^10^. For instance, miR-155 and miR-21 are up-regulated in breast cancer, but miR-91 has been found to be down-regulated in breast cancer. Other miRNAs can be related with more than one type of cancer. Many recent studies show that miR-145 is a tumour-suppressive miRNA, that is downregulated in several cancer types, including bladder cancer^11^, colon cancer^12^, breast cancer^13^, ovarian cancer^14^ and prostate cancer (PCa)^5^.

For PCa, miR-145 is a well-characterised tumour-suppressor with an important regulatory role since it can protect from cancer cell invasion and metastasis^15^. miRNAs have been reported to act as fingerprints of a disease, including PCa, making them a promising tool as a biomarker. Conventionally, technologies used for miRNA study include northern blotting^16^, *in situ* hybridization^17^, quantitative polymerase-chain reaction^18^ and miRNA microarrays. All these techniques are powerful but laborious and hence restricted to central laboratories. Consequently, there is a pressing need to develop simple and sensitive techniques to quantify levels of miRNAs in a portable and inexpensive way^9^,^19^.

To take up such a challenge, electrochemical biosensors find their right application, thanks to their varied advantages such as specificity, portability and low cost^20^. There is a handful of reports on detection of miRNAs using electrochemical approaches. Electrochemical impedance spectroscopy (EIS) using redox markers has been previously used to quantify target miRNA hybridisation down to femtomolar levels with complex amplification techniques^19^,^21^. Other electrochemical techniques such as differential pulse voltammetry (DPV) have also been used to detect miRNAs with a limit of detection (LOD) over picomolar levels^22^.

However, the developed biosensors reported lack simplicity in their fabrication process. To address this issue, we report the development of a simple and sensitive dual mode electrochemical detection platform for miRNA using peptide nucleic acids (PNA) as probes. PNA was used since it presents many advantages such as neutral charge and higher stability than its biological counterparts. Also, the PNA/miRNA duplex with mismatches is less stable than a DNA/miRNA duplex with the same mismatches^23^,^24^. Furthermore, a simple and powerful amplification strategy using positively charged gold nanoparticles (AuNPs) has been employed. EIS was used without redox markers to monitor the changes in the dielectric properties of the bilayer through capacitance changes. Such an approach further simplifies the detection strategy making it suitable for easy integration into the system. Our group previously reported the development of a capacitive and potentiometric sensor for DNA detection based on the same strategy^25^. In this work, the detection strategy was applied to detect miR-145 sequence using EIS. In order to reduce false positives, a complementary detection technique was developed on the same sensors using square wave voltammetry for validation of the signals. Such a step was performed by exploiting the availability of AuNPs on the surface with PNA/miRNA duplex to attach thiolated ferrocene in order to provide a voltammetric detection using square wave voltammetry (SWV). With the developed dual detection system on the same sensor, a LOD of 0.37 fM was achieved with a wide dynamic range from 1 fM to 100 nM.

## Results and Discussion

### Characterisation of PNA-based sensor

**Figure 1:**
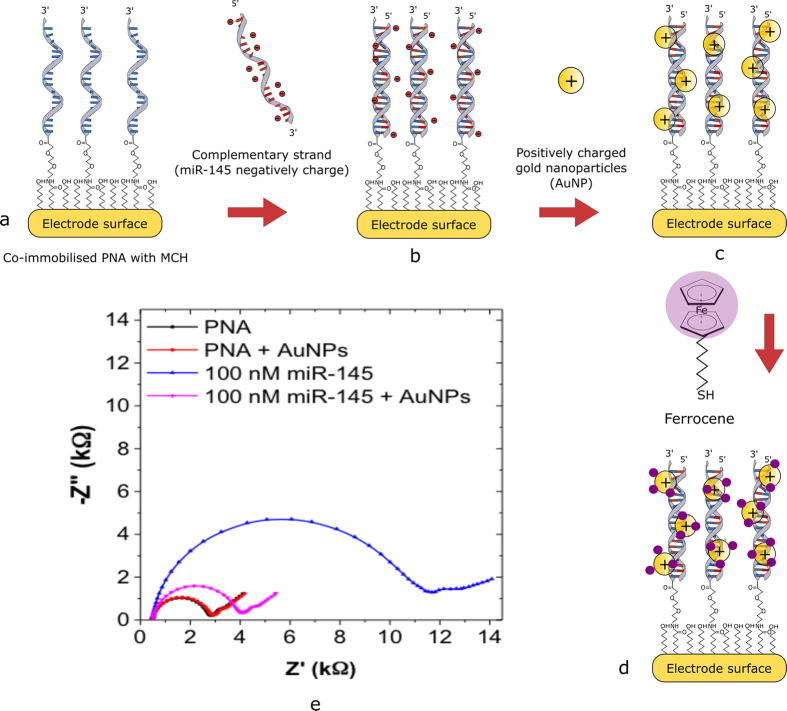
(**a**) Typical Cole-Cole plot obtained with PNA-based sensor before and after hybridisation with 1 fM miR-145 (black and blue respectively) and AuNP attachment (pink). A zoom-in version of Cole-Cole plot shows the changes in capacitance upon a molecular binding event. (**b**) Calibration curve and data points represent average changes in capacitance from a minimum of three independent samples upon binding with AuNPs at different concentration of miR-145.

Figure 1 provides a schematic of the detection strategy (a–d) and a Nyquist plot illustrating changes in the electrical properties of the gold electrode during the modification process (e). EIS in a Faradaic mode was used to characterise any molecular binding event. A relatively low *R*_ct_ of 2.9 kΩ was recorded due to physical barrier imposed by the presence of the self-assembled monolayer (SAM) comprised of co-immobilised uncharged PNA and 6-mercapto 1-hexanol (MCH) on the surface. Initially, the PNA immobilised biosensor was tested with positively charged AuNPs to study non-specific interactions. As seen in Fig. 1 a negligible change in the *R*_ct_ (ca. 2.5%) of the system was observed, indicating that no AuNPs bind to the system. The PNA-based biosensor was then incubated with 100 nM miR-145 for 30 minutes, where upon hybridisation a significant increase in *R*_ct_ from 2.9 kΩ to 11.9 kΩ was recorded. An increase of *R*_ct_ is attributed to the formation of a PNA/miR145 duplex, making the electrode surface highly negatively charged and creating an electrostatic barrier to the negatively charged redox marker in the measurement solution.

Finally, AuNPs modified with poly(ethylenimine) (PEI) were added, which electrostatically bind to the PNA/miR145 duplex because of the positive charges of the amine groups on the modified AuNPs, resulting in a decrease in the *R*_ct_ of the system from 11.9 kΩ to 4 kΩ. Such a decrease could be attributed to the screening of negative charges of the PNA/DNA duplex with positively charged AuNPs. It is worth mentioning that the *R*_ct_ of the system does not return to its initial value. Such an observation could be attributed to the fact that although AuNPs are screening the charges, the mass loading effect still causes an increased physical barrier to the redox couple.

**Figure 2:**
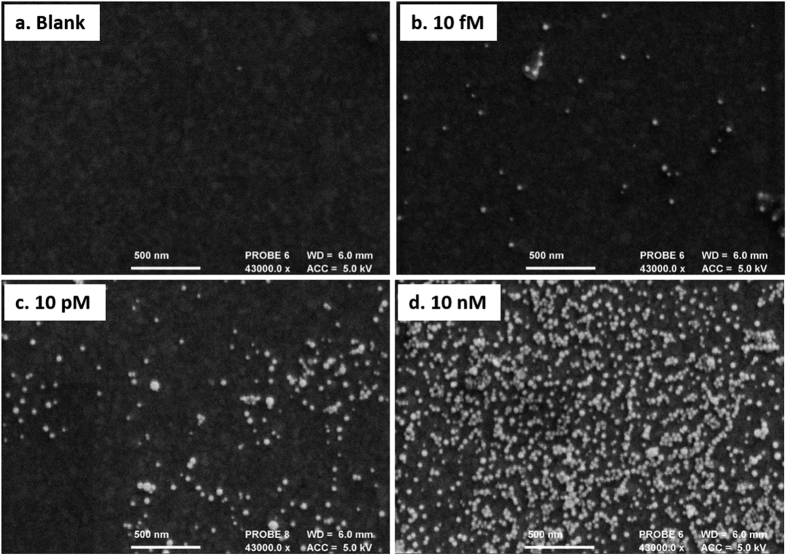
SEM micrograph showing the distribution of AuNPs after hybridisation with (**a**). Blank, (**b**): 10 fM, (**c**): 10 pM and (**d**): 10 nM of mir-145.

The molecular interaction after incubation with AuNPs was also examined using scanning electron microscopy (SEM) – see Fig. 2. For the blank sample (i.e. no miRNA), no AuNPs are observed showing that there is almost no non-specific interactions between the AuNPs and the PNA SAM. Upon increasing concentrations of target miR-145, more nanoparticles are visible in the samples. It is worth mentioning that aggregation of AuNPs is significantly observed at high concentrations (Fig. 2d: 10 nM).

**Figure 3:**
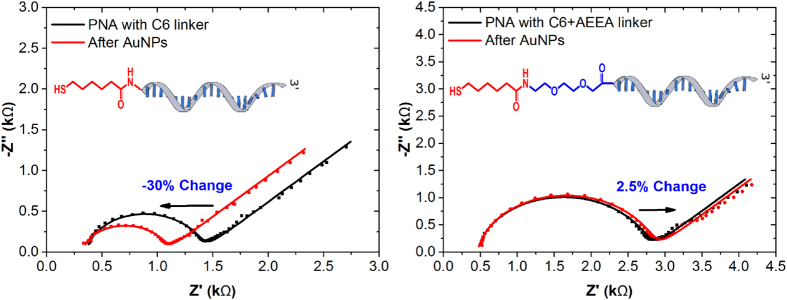
Faradaic EIS plots of PNA based biosensor. (Left) Represents C6-Linker modified PNA as a probe for biosensor fabrication and the change in Rct after incubating with AuNPs. (Right) Represents C6-AEEA Linker modified PNA as a probe for biosensor fabrication and the change in Rct after incubating with AuNPs.

In our previous work, we reported small signal changes in capacitance upon hybridisation with complementary DNA due to the length of the polyethylene glycol (PEG)-like linker with PNA probe^25^. Therefore, in order to increase the signal change with hybridisation, we studied the effect of the size of the PNA linker. A C6 thiol linker can be used in place of the C6-AEEA (where AEEA is a 8-amino-3,6-dioxaoctanoic acid glycol linker) linker as a PNA modification that can be co-immobilised on the gold surface (see Fig. 3). By reducing the linker length from C6-AEEA to C6, a significant variation in the capacitance of over 5% was observed upon hybridisation with the complementary miRNA strand. However, what was more important in this particular electrochemical platform development, was to test its selectivity efficiency. For the same, the biosensor was constructed using both modifications of PNA (C6 and C6-AEEA) with MCH on the gold surface. Electrodes were subsequently tested with just AuNPs without any hybridisation. EIS in Faradaic mode was used to monitor the changes in *R*_ct_. Figure 3 illustrates impedance plots recorded from the EIS experiment. A significant non-specific interaction of around 30% was observed as a result of the reduction in *R*_ct_ after incubation of PNA-based biosensor with C-6 linker with AuNPs. A decrease in *R*_ct_ is a result of attachment of positively charged AuNPs to the electrode surface which consequently results in a decrease in the resistance to negatively changed redox couple in the solution.

Such an observation indicates that the SAM formed on the gold surface is not efficient enough to prevent non-specific interactions. As compared to the C6 linker, when a C6-AEEA linker was employed, a significant reduction in the non-specific interaction to less than 3% was observed showing an enhanced anti-fouling efficiency, thanks to the PEG-like linker. Such an enhanced antifouling effect could be predominantly due to the creation of a hydration layer by the presence of the AEEA. PEG coatings have been previously reported to prevent non-specific binding as each PEG ether group can H-bound with two water molecules creating a strong hydration shell^26^. Such a hydration layer prevents proteins and other charged molecules to penetrate through the water layer and adsorb on the surface^27^,^28^,^29^. As a result, only C6-AEEA modification of PNA probes was used in our sensors from here on.

### Capacitive Sensing

**Figure 4:**
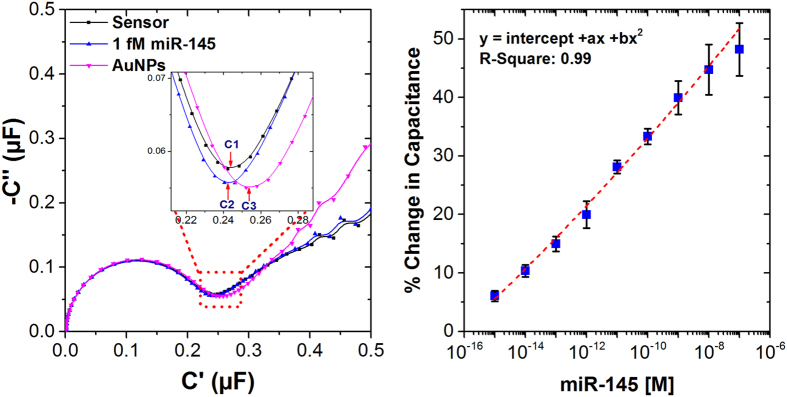
(**a**) Typical Cole-Cole plot obtained with PNA-based sensor before and after hybridisation with 1 fM miR-145 (black and blue respectively) and AuNP attachment (pink). A zoom-in version of Cole-Cole plot shows the changes in capacitance upon a molecular binding event. (**b**) Calibration curve and data points represent average changes in capacitance from a minimum of three independent samples upon binding with AuNPs at different concentration of miR-145.

The detection of different concentrations of miR-145 was successfully monitored using EIS in non-Faradaic mode by estimating the changes in the capacitance upon a molecular binding event^25^,^30^. The main advantage of using a non-Faradaic mode EIS measurement technique is the simple and easy execution of the measurements compared to any labelled biosensors. In order to evaluate the capacitance of the system, a complex capacitance was defined (see supplementary information, S-1). From the capacitance data, Cole-Cole capacitance plots can be obtained (see supplementary information, Figure S-1). With these plots, the diameter of the obtained semicircle corresponds the capacitance of the system. Figure 4 (left) depicts an example of *Cole-Cole* plots obtained from non-Faradaic EIS measurements of the electrode modified with a PNA sequence which was used to detect 1 fM of miR-145. An initial capacitance of 0.243 μF was observed with PNA-based sensor (*C*1) which upon hybridisation with 1 fM of target miR-141, decreased to 0.241 μF (*C*2), leading to a change of −0.82%. As reported earlier, only a slight variation in capacitance is observed upon miR-145 hybridisation^25^. The probable reason for this small signal is that the PNA has a linker which is a derivate of PEG (AEEA, C_21_H_23_NO_6_) with a length of 1.4 nM. As a result, the binding event is happening far away from the surface and well above the Debye length of the system, leading to screening of the DNA charges by the electrolyte. However, when incubated with positively charged AuNPs, a significant increase in capacitance was observed due to disruption of the electrochemical double layer. From Fig. 4 (left), incubating with AuNPs leads to a capacitance increase to 0.254 μF (*C*3), leading to more than 6 folds increase due to AuNPs amplification with a percentage change of +5.4%. Such an amplified response could be attributed to the molecular interactions with AuNPs near the electrode surface where redistribution of the solvent molecules and of ions is initiated, which causes changes in the electrochemical double layer.

The fabricated PNA-based biosensor was tested with a wide range of miR-145 concentrations from 1 fM to 100 nM in 10 mM PB pH 7.4, where an increase in the change in the capacitance upon AuNP incubation was observed with increasing miRNA concentrations. It is worth mentioning that the electrode was used only once with each concentration. After 30 minutes incubation at each concentration, the variation in capacitance was proportional to the amount of captured miR-145. Based on the variation of capacitance corresponding to attachment of AuNPs, a calibration curve was obtained as a function of miR-145 concentration. The biosensor demonstrated an excellent response (Fig. 4, right). The sensor could potentially detect miR-145 down to 1 fM with a nearly 6% change. The obtained dose-response for the specific capture of miR-145 presented in Fig. 3 follows indeed a typical second order polynomial fit (quadrant equation) with a root mean square value (R-Square) of 0.99 and an intercept of 5.60 + 0.58%. A very low limit of detection (LOD) of 0.37 fM was calculated using 4 independent samples as described by Armbruster *et al*.^31^. The demonstrated LOD with the current detection technique is better or comparable to the state-of-art technologies reported for electrochemical detection of miRNAs. Furthermore, the electrochemical sensor presents a simple and inexpensive detection strategy as compared to using complex fabrication steps or expensive enzymes (see Table 1).

### Voltammetric Biosensor

With successful application of a simple amplification strategy to detect miR-145 using capacitance measurements, the setup was explored for additional applications. One such application explored the availability of AuNPs on the surface of the electrode^32^. Formisano *et al*. demonstrated the use of AuNPs on the electrode surface as a possible platform to immobilise thiolated ferrocene molecules^33^; such a modified AuNP with ferrocene was used as a redox indicator by monitoring the oxidation peaks of ferrocene.

**Figure 5:**
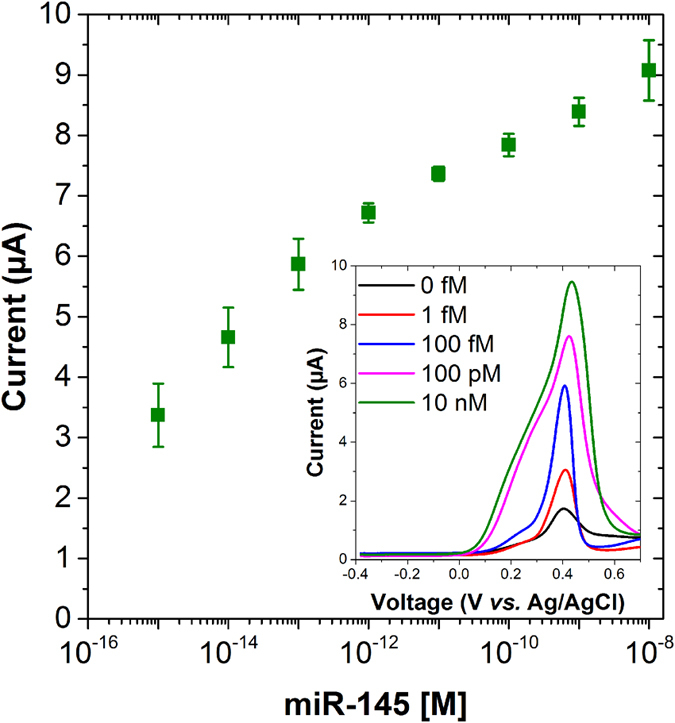
Dose response. Data points represent average ferrocene peaks recorded using square wave voltammetry (SWV) from three independent samples on the binding of thiolated ferrocene with AuNPs at different concentration of mir145 (left). Inset shows the SWV curves recorded at different miR-145 concentrations.

Since detecting ultra-low levels of miRNAs can introduce false positives in the signal, a complementary voltammetry technique was developed in the present study by using thiolated ferrocene molecules (6-(ferrocenyl)hexanethiol), which will serve as a validation technique to confirm the binding event. After specific interaction of AuNPs with PNA/miR-145 duplex, 50 μM thiolated ferrocene was flowed through the system, allowing the thiolated ferrocene to covalently bind to the free available spaces of AuNPs. The PNA-based biosensor was tested with a wide range of miR-145 concentrations from 1 fM to 100 nM in 10 mM PB pH 7.4, where an increasing oxidation peak of ferrocene was observed (Fig. 5) after the final step of thiolated ferrocene flow using square wave voltammetry (SWV).

From Fig. 5, it can be seen that the peak current increases for increasing miR-145 from 1 fM to 1 pM, after which the peak current starts saturating, resulting in similar peak currents. Such saturation could be due to aggregation of AuNPs at a higher miR-145 concentration resulting in decreased charge transfer. A similar effect could be seen in the SWV recording (inset Fig. 5) where as we move towards higher miR-145 concentration, a wider ferrocene peak was observed which could be attributed to the non-homogeneous assembly of ferrocene near the electrode surface. As a result, two processes could be seen due to diffusion effects of electrons; one is the fast oxidation response due to presence of some amount of ferrocene closer to the electrode surface and later a relatively slower response due to presence of ferrocene further away from the gold electrode surface^34^. The ferrocene peaks are also affected by the environment where, for example, the presence of amine groups could shift the peaks towards more positive potential^35^. It is worth mentioning that a high background current of nearly 1 μA was recorded. Such a current could be due to two probable reasons. Firstly, due to the non-specific interaction of ferrocene to the electrode surface and secondly, due to the presence of even a small amount of AuNPs due to non-specific interaction which could be used as a platform where ferrocene can attach. Nevertheless, when the blank measurement was compared with the lowest miR-145 concentration used, a six time higher ferrocene peak current was recorded (Fig. 5).

With the application of ferrocene, unlike any other reported biosensor for miRNA detection, a simple and powerful dual detection technique could be performed where the signals from ferrocene can be used as an alternative detection technique to validate the results obtained from the capacitive measurements. As a result, more reliable data could be generated for any molecular event and thereby, reducing overall false positives.

### Selectivity Study

**Figure 6:**
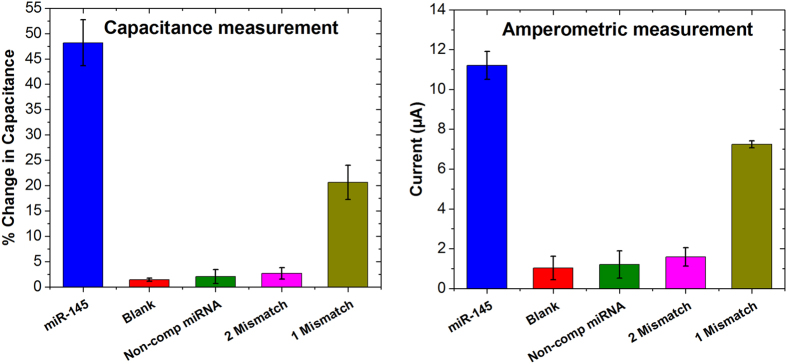
Selectivity study with mismatched miRNA sequences. All the RNAs concentration were 100 nM.

The developed PNA-based biosensor was challenged with stringent controls in order to investigate the selectivity of the sensor. For the study, 100 nM concentration of miR-145 was compared with 100 nM of RNA sequence with 1 mismatch, 2 mismatches, and a non-complementary RNA. The results from both capacitance measurement and SWV are presented in Fig. 6.

In both types of measurements, the signal change observed with 100 nM of RNA sequence with 2 mismatches and a non-complementary RNA showed negligible signal change when compared with 100 nM miR-145. However, with 1 mismatch sequence, a noteworthy change in the signal was observed with both detection techniques. Such an interaction could be due to the choice of the mismatch. As shown in Table 2, the mismatch is in the middle of the sequence, which is considered the most stable position in PNA/DNA or PNA/miRNA hybridisation^36^. Nevertheless, the signal change observed was still 50% lower than what was observed with miR-145. The non-specific interaction observed with mismatched sequences could be reduced by adding another parameter of temperature. By using a temperature close to the melting point of the PNA/miR-145 duplex, the non-specific binding of mismatches can be greatly reduced.

## Conclusions

In the current work, a highly sensitive dual-mode electrochemical biosensor with a simple AuNP amplification for the detection of miR-145 is presented. It has been shown how AuNPs can disrupt the dielectric biolayer of the sensor, leading to significant changes in the capacitance of the system. Also, a complementing detection technique was demonstrated by exploiting the availability of AuNPs on the surface with PNA/miRNA duplex; thiolated ferrocene was used to provide a voltammetric detection using SWV. A dual-mode approach can be used as a validation detection technique to compare the sensor’s performance and reduce inconsistencies in output signals. With the current PNA-based system, a wide dynamic range from 1 fM to 100 nM with a LOD of 0.37 fM was achieved. Of significant importance is that the detection technique reported can easily be expanded into arrays for the parallel screening of panels of DNAs or miRNAs.

## Methods

### Electrochemical setup

The experiments were carried out with a CompactStat potentiostat (Ivium Technologies, The Netherlands) and a three-electrode cell system: Ag/AgCl (KCl) reference electrode (BASi, USA) connected via a salt bridge filled with 10 mM phosphate buffer (PB, pH 7.4), Pt counter electrode (ALS, Japan) and gold working electrode (2.0 mm diameter, CH Instruments, USA). The non-Faradaic impedance spectrum was conducted in 10 mM PB (pH 7.4) measurement buffer over a frequency range from 100 kHz to 100 mHz, with a 10 mV a.c. voltage superimposed on a bias d.c. voltage of 0 V with respect to the open circuit potential^25^. The Faradaic impedance spectrum was conducted in 10 mM PB (pH 7.4) measurement buffer containing 10 mM of the ferro/ferricyanide [Fe(CN)_6_]^3−/4−^ redox couple (potassium hexacyanoferrate II/III, Sigma, UK) over a frequency range from 100 kHz to 100 mHz, with a 10 mV a.c. voltage superimposed on a bias d.c. voltage of 0.2 V *vs.* Ag/AgCl (which corresponds to the formal potential of the redox couple). The square wave voltammetry technique (SWV) was used to monitor the oxidation peaks of the ferrocene attached to the AuNPs. SWV was performed in 10 mM PB (pH 7.4) in the potential range from −0.4 V to 0.7 V *vs.* Ag/AgCl with conditioning time of 120 s, modulation amplitude of 20 mV and frequency of 50 Hz.

For characterisation of AuNPs, stock AuNPs were diluted ten times in MilliQ water, and optical absorption spectra were measured using a UV–vis spectrophotometer (UV-1800/TMSPC-8, Shimadzu, UK) using a 8-cell quartz cuvette with 150 μL sample size. The wavelength for absorbance analysis was scanned from 800 nM to 400 nM.

### Oligonucleotides

HPLC purified synthetic oligonucleotides were purchased from Sigma-Aldrich, UK in lyophilised form, while PNA probe sequences were purchased from Cambridge Research Biochemical, UK. The sequences are shown in Table 2.

### Biosensor fabrication

Gold working electrodes were cleaned using a protocol previously described in Jolly *et al*.^25^ electrodes were first mechanically polished for 2 minutes with 1 μM diamond solution (Buehler, USA) and thereafter for 5 minutes with 50 nM alumina slurry (Buehler, USA) on polishing pads (Buehler, USA). In between each step, 10 minutes sonication and rinsing in Milli-Q water were performed to remove any remaining particle residues. Additionally, electrodes were exposed to chemical cleaning using piranha solution (3 parts of concentrated H_2_S0_4_ with 1 part of H_2_0_2_) for 10 minutes. Electrodes were then rinsed with ultra-pure water and thereafter electrochemically cleaned in 0.5 M H_2_S0_4_ (Sigma-Aldrich, UK) by scanning the potential between the oxidation and reduction of gold, 0 V and +1.5 V *vs.* an Ag/AgCl reference electrode, for 50 cycles until no further changes in the voltammogram were noticed. Finally, the electrodes were rinsed with Milli-Q water.

Clean gold electrodes were then co-immobilized with a thiolated ssPNA probe sequence and 6-mercapto-1-hexanol (MCH, Sigma-Aldrich, UK) in 50% dimethyl sulfoxide (DMSO, Sigma-Aldrich, UK), 50% ultra-pure water (v/v) immobilisation solution for 16 h in a humidity chamber. An optimized ratio of 1:5 was adopted from the literature^25^. After immobilisation, electrodes were rinsed with surplus MilliQ water to remove any unattached thiols. In order to ensure complete thiol coverage of the gold surface, the electrodes were backfilled with 1 mM MCH for 1 hour. Electrodes were then rinsed with MilliQ water and placed in the measurement buffer for 1 hour to stabilise the SAM. The functionalised electrodes were then used to detect target miR-145.

Preparation of positively charged AuNPs was adopted as reported by Kim *et al*.^36^. Briefly, an optimised ratio of hydrogen tetrachloroaurate (HAuCl_4_, Sigma-Aldrich, UK) and branched poly-(ethylenimine) (PEI, MW ~ 25 kDa, Sigma-Aldrich, UK) was mixed overnight using vigorous stirring to produce AuNPs of average size of 20 nM. Before the experiments, the AuNPs were washed three times using centrifugation and re-dispersion in Milli-Q water. Optical characterisation of PEI coated AuNPs was performed using a spectrophotometer where a characteristic absorbance peak at 523 nM (see supplementary information S-2) was observed, which is similar to the previously reported results for AuNPs with 20 nM size^37^,^38^.

For the binding studies, different concentrations of target miR-145, non-complementary miRNAs and 1- or 2-mismatched miRNA sequences were prepared in 10 mM PB, pH 7.4.

### Surface Characterisation

50 nM gold coated SPR gold chips, supplied from Reichert Technologies were used for studying the reaction. SPR chips were modified with PNA probes as described in the biosensor fabrication section. Binding study with 0 fM, 10 fM, 10 pM and 10 nM miR-145 was performed on the chip followed by incubation with AuNPs. Thereafter, the chips were dried with N_2_ flow and were left in vacuum overnight. Surface characterization of SPR chips were performed using a scanning electron microscopy (JSM-6480Jeol, Japan) at 43000X magnification with acceleration voltage of 5 kV to acquire SEM images.

## Additional Information

**How to cite this article**: Jolly, P. *et al*. Highly sensitive dual mode electrochemical platform for microRNA detection. *Sci. Rep.* **6**, 36719; doi: 10.1038/srep36719 (2016).

**Publisher’s note:** Springer Nature remains neutral with regard to jurisdictional claims in published maps and institutional affiliations.

## Supplementary Material

Supplementary Information

## Figures and Tables

**Table 1:** Comparison with the reported state-of-art electrochemical platforms for miRNA detection.

Detection Method	Electrode Surface	MicroRNA	LOD	Reference
CV & EIS	Gold electrode	miR-21	3.96 pM	Zhou *et al*.^39^
DPV & EIS	Gold electrode modified with AuNP	miR-21	6 fM	Meng *et al*.^40^
CV & DPV	Gold electrode	miR-21	45 fM	Xia *et al*.^41^
EIS	Gold electrode and duplex specific nuclease as an amplification tool	let-7a, let-7b, let-7c	1 fM	Ren *et al*.^42^
DPV & EIS	Gold electrode modified with AuNP	miR-159a	3.5 fM	Wang *et al*.^43^
CV	Gold electrode	miR-21	3 fM	Liu *et al*.^44^
DPV & EIS	Gold electrode	let-7a, let-7b, let-7c	99.2 fM	Peng *et al*.^45^
CV, DPV & EIS	Gold electrode modified with AuNPs	miR-21	0.17 fM	Wang *et al*.^46^
DPV	Gold electrode modified with AuNP	miR-139a	1.7 fM	Zhou *et al*.^47^
DPV	Gold electrode modified with AuNP	miR-21	0.36 fM	Li *et al*.^48^
**EIS & SWV**	**Gold Electrode**	**mir145**	**0.37** **fM**	**This study**

CV: Cyclic voltammetry; DPV: Differential Pulse Voltammetry; EIS: Electrochemical Impedance Spectroscopy; SWV: Square Wave Voltammetry.

**Table 2:**
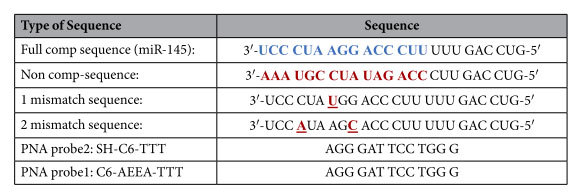
miRNA sequences and PNA probes used in this work.

The full complementary sequence in the first row corresponds to the mir-145 sequence. AEEA is a glycol linker of nine atoms (8-amino-3,6-dioxaoctanoic acid).
