# Corrigendum: Highly sensitive dual mode electrochemical platform for microRNA detection

**DOI:** 10.1038/srep41110

**Published:** 2017-01-23

**Authors:** Pawan Jolly, Marina R. Batistuti, Anna Miodek, Pavel Zhurauski, Marcelo Mulato, Mark A. Lindsay, Pedro Estrela

Scientific Reports 6: Article number: 3671910.1038/srep36719; published online: 11 08 2016; updated: 01 23 2017

In this Article, the legend of Figure 1 is a duplication of the legend of Figure 4:

“(a) Typical Cole-Cole plot obtained with PNA-based sensor before and after hybridisation with 1 fM miR-145 (black and blue respectively) and AuNP attachment (pink). A zoom-in version of Cole-Cole plot shows the changes in capacitance upon a molecular binding event. (b) Calibration curve and data points represent average changes in capacitance from a minimum of three independent samples upon binding with AuNPs at different concentration of miR-145.”

Should read:

“Schematic of the biosensor construction: (a) Immobilized PNA probes; (b) Capture of target oligo (DNA/miRNA); (c) Electrostatic interaction of positively charged AuNPs with PNA/DNA or PNA/miRNA hybrid and finally (d) Attachment of thiolated ferrocene to attached AuNPs. (e) EIS characterisation of binding events using PNA based biosensor: the black curve represents PNA based biosensor, red curve represents non-specific binding of AuNPs, blue curve represents the hybridisation of mir145 with PNA probes and finally magenta curve represents the electrostatic interaction of AuNPs with PNA/mir145 duplex.”

