# Supplementary material for: Highly sensitive dual mode electrochemical platform for microRNA detection
Source: Sci Rep. 2016 Nov 8;6:36719. doi: 10.1038/srep36719 (PMC5099858; doi:10.1038/srep36719)
Supplement: Supplementary Information [file srep36719-s1.pdf]

<u>**Supplementary Information**</u>

Highly sensitive dual mode electrochemical platform for microRNA detection

Pawan Jolly, Marina R. Batistuti, Anna Miodek, Pavel Zhurauski, Marcelo Mulato,

Mark A. Lindsay, Pedro Estrela

S-1: EIS measurement in non-Faradaic mode

The detection of different concentrations of miR-145 was successfully monitored using EIS in non-Faradaic mode by estimating the changes in the capacitance upon a molecular binding event[1, 2]. In order to evaluate the capacitance of the system, a complex capacitance was defined as:

$$C^* = -\frac{Z''}{\omega|Z|^2} - j\frac{Z'}{\omega|Z|^2} = C' + jC'' \qquad (1)$$

Where *C'* corresponds to the real part of the capacitance and *C"* corresponds to the imaginary part of the capacitance. *Z'* and *Z"* are the real and imaginary components of the measured impedance, respectively, and ω=2πf is the angular frequency of the measurement. From the capacitance data, *Cole-Cole* capacitance plots can be obtained. With these plots, the diameter of the obtained semicircle corresponds the capacitance of the system. Figure S-2 depicts an example of non-Faradaic EIS measurement of the electrode modified with a PNA sequence which was used to detect 1 fM of miR-145 (left is a Nyquist plot and right is calculated capacitance from EIS data, diameter of the semicircle of which gives an estimate of the capacitance of the system). An initial capacitance of 0.243 μF was observed with PNA-based sensor (C1) which changed to 0.241 μF after incubating with 1fM miR-145 (C2), leading to a change of -0.82%. However, after incubating with AuNPs, the capacitance increased to 0.254 μF (C3), leading to change of +5.4%.

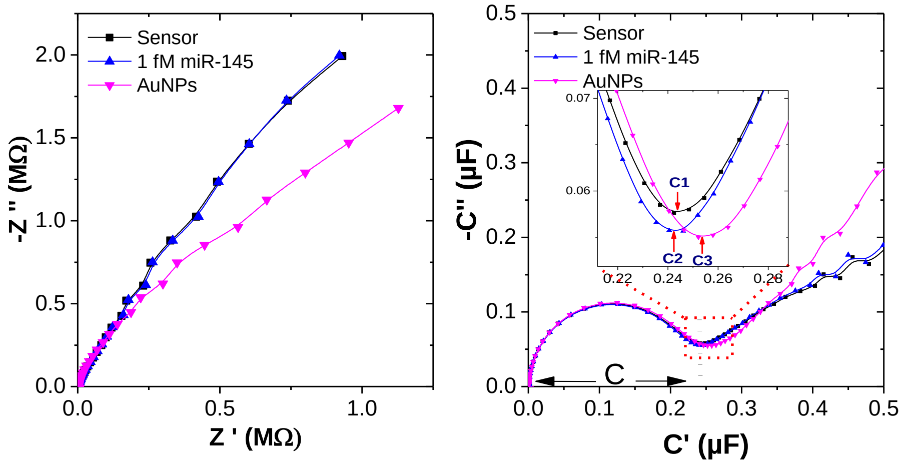

Figure S-1 - ***Figure showing a Nyquist plot (left) representing the impedance of the system. On the right is a Cole-Cole plot representing the capacitance of the system calculated from impedance data. A zoom in version of Cole-Cole plot below depicting the changes in capacitance with 1 fM miR-145.***

S-2: Optical characterisation of PEI coated gold nanoparticles

Preparation of positively charged AuNPs was adopted as reported by Kim et al.[3]. Briefly, an optimised ratio of hydrogen tetrachloroaurate ($HAuCl_4$, Sigma-Aldrich, UK) and branched poly-(ethylenimine) (PEI, MW~25 kDa, Sigma-Aldrich, UK) was mixed overnight using vigorous stirring to produce AuNPs. Before experiments, the AuNPs were washed three times using centrifugation and re-dispersion in Milli-Q water. For characterisation of AuNPs, stock AuNPs were diluted ten times in MilliQ water, and optical absorption spectra were measured using a UV–vis spectrophotometer from Shimadzu, UK (Shimadzu UV-1800 / TMSPC-8) using a 8-cell quartz cuvette with 150 µL sample size. The wavelength for absorbance analysis was scanned from 800 nm to 400 nm. Optical characterisation of PEI coated AuNPs were performed using spectrophotometer where a characteristic absorbance peak at ~523 nm (see Figure S-2) was observed, which are similar to the previously reported results for AuNPs with size of ~20 nm[3, 4].

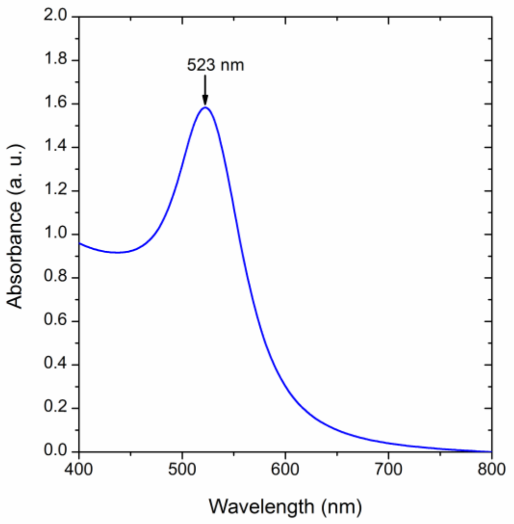

Figure S-2 - ***UV–Vis spectra of PEI coated AuNPs***

References

1. Jolly, P. *et al.* Label-free impedimetric aptasensor with antifouling surface chemistry: A prostate specific antigen case study. *Sens Actuators B.* **209**, 306-312 (2015).
2. Jolly, P., Wong, L., Miodek, A., Lindsay, M.A. & Estrela, P. A simple and highly sensitive electrochemical platform for detection of MicroRNAs. *Proc IEEE Sensors.* 1-4 (2015).
3. Kim, K., Lee, H.B., Lee, J.W., Park, H.K. & Shin, K.S. Self-assembly of poly (ethylenimine)-capped Au nanoparticles at a toluene− water interface for efficient surface-enhanced Raman scattering. *Langmuir.* **24**(14), 7178-7183 (2008).
4. Zhang, Xiao-Dong, *et al.* Size-dependent radiosensitization of PEG-coated gold nanoparticles for cancer radiation therapy. *Biomaterials.* **33(**27), 6408-6419 (2012).
